# Comparative Study of Crude and Wine-Processing *Corni Fructus* on Chemical Composition and Antidiabetic Effects

**DOI:** 10.1155/2019/3986964

**Published:** 2019-12-02

**Authors:** Huailong Bi, Dou Niu, Chen Guo, Jia Li, Xue Chen, Yuan Zhang, Shaojun Wang, Jiqing Bai, Xing Li, Jiefang Kang

**Affiliations:** ^1^Key Laboratory of the Ministry of Education for Medicinal Resources and Natural Pharmaceutical Chemistry, National Engineering Laboratory for Resource Developmentof Endangered Crude Drugs in Northwest of China, College of Life Sciences, Shaanxi Normal University, Xi'an, Shaanxi 710062, China; ^2^Shaanxi University of Chinese Medicine, Xianyang, Shaanxi 712046, China

## Abstract

Wine processing is a specialized technology which involves sautéing crude herbal medicine using Chinese rice wine. Herein, we identified the changes in chemical profiles and antidiabetic effects of *Corni Fructus* (CF) after wine processing in high-fat diet (HFD) streptozotocin- (STZ-) induced diabetic mice. A novel high-efficiency method for simultaneously quantifying gallic acid, 5-hydroxymethylfurfural, morroniside, loganin, sweroside, and cornuside by UPLC was developed, and validating crude and wine-processing CF was done for the first time. Mice were randomly divided into the following groups and orally given different solutions for 4 weeks: normal group (NC, 0.4% (w/v) CMC-Na), model group (DM, 0.4% (w/v) CMC-Na), crude CF group (CP, 3.87 g/kg), and wine-processing CF group (PP, 3.87 g/kg) followed by HFD and multiple subcutaneous injection of STZ (40 mg/kg) to induce the diabetes model except the NC group. Biochemical indexes (body weight, fasting blood glucose level, lipid level, insulin, and free fatty acid) and other parameters involving liver toxicity were measured with commercial kits and immunohistochemical method. Comparative studies on pharmacology showed that the crude extracts possess higher efficacy on hypoglycemia and hypolipidemia, while wine-processing products exhibit better effects on liver preservation. Our data suggested that wine processing was recommended when CF was used for protecting the liver; however, crude products should be used as antidiabetic drugs.

## 1. Introduction

Type 2 diabetes mellitus (T2DM), also known as noninsulin-dependent DM, affects more than 90% of the diabetics and/or leads to serious lipid and protein metabolism disorders, with a characteristic of hyperglycemia and insulin resistance (IR), which is caused by impaired insulin secretion [1]. The current prevalence of T2DM is associated with a high calorie diet and a sedentary behavior [2], and metformin has become the preferred first-line prescribed oral hypoglycemic worldwide to treat T2DM. Metformin can improve IR, notably in the muscle and liver, and reduce hepatic glucose. However, the applications are limited by adverse effects. Metformin alone cannot completely control blood sugar, especially when the disease becomes more severe [3–5]. Furthermore, it cannot facilitate insulin secretion, and high concentrations of metformin could stimulate intestinal cells [6, 7]. Therefore, new drugs with low toxicity and high antidiabetic efficiency from natural resources have been attracted attention by researchers [8].

*Corni Fructus* (CF), the dried fruit of *Cornus officinalis* Siebold et Zuccarini., also known as “Shan Zhu Yu” in Chinese, is a tonic capable of nourishing the liver and kidney, treating impotence, removing internal heat, and so on [9]. Beyond treating DM-induced reproductive dysfunction in the formulations of traditional Chinese medicine (TCM), Liu Wei Di Huang tea pills [10], there is accumulating evidence suggesting that CF is widely used for the treatment of DM. Currently, CF has been extensively investigated in phytochemistry. The main active components, including gallic acid, 5-hydroxymethylfurfural (5-HMF), morroniside, sweroside, loganin, and cornuside, possess different preferable biological activities. Gallic acid could protect against oxidative stress-induced damage in the diabetic state [11]. Loganin had a protective effect against hepatic oxidative stress [12]. Morroniside showed effective anti-inflammatory properties in AMI rats [13]. Sweroside could ameliorate liver injury by decreasing oxidative damage and inhibiting the production of proinflammatory kinases [14, 15]. Cornuside has been reported to possess immunomodulatory and anti-inflammatory activities [16, 17]. 5-HMF helped preventing diabetes-associated vascular diseases [18]. Previous studies demonstrated that CF exhibits immune-regulating function, antishock effects, and protective effects on experimental diabetic nephropathy [19]. It also been reported that loganin, morroniside, and gallic acid isolated from the CF had a synergistic effect of hypoglycemic [20].

The processing method (PaoZhi in Chinese) plays a vital role in detoxification of TCM, which can decompose the toxic components into less or nontoxic derivatives, stabilize the effective ingredients, and improve the curative effects of TCM [21]. There are various processing methods for TCM, and wine processing, which is sautéing with rice wine, is one of the most popular technologies for herb processing. Either crude or wine-processing products of CF were commonly used clinically. Here, we have evaluated the antidiabetic activity of the crude and wine-processing CF since the changes in component content and different functions are caused by processing.

## 2. Materials and Methods

### 2.1. Chemicals and Reagents

A high-fat diet (HFD) D12492 (60 kcal% Fat) (Research Diets Inc., NJ, USA) was purchased from Yes Service Biotech (Shanghai, China). Metformin tablets were purchased from Bristol-Myers Squibb (China) Investment Co., Ltd. (Shanghai, China). Sodium carboxymethylcellulose (CMC-Na) was purchased from Anhui Sunhere Pharmaceutical Excipients Co., Ltd. (Huainan, China). Test kits of serum alanine aminotransferase (ALT), aspartate aminotransferase (AST), total cholesterol (TC), serum triglycerides (TG), low-density lipoprotein cholesterol (LDL-C), and high-density lipoprotein cholesterol (HDL-C) were purchased from Beijing BeihuaKangtai Clinical Reagent Company (Beijing, China). The commercial kits used for enzyme-linked immunosorbent assay (ELISA) were obtained from Tianjin Anoric Bio-technology Co., Ltd. (Tianjin, China). Streptozocin (STZ) injections were purchased from Sigma Chemical Co. (St Louis, MO, USA). One-touch glucometer was obtained from Hermano Technology Co., Ltd (Shenzhen, China). The 0.22 *μ*m polyvinylidene fluoride (PVDF) millipore syringe filter used was purchased from Jinteng Co., Ltd. (Tianjin, China).

Chinese rice wine (Huangjiu) as the solvent was acquired from Shaoxing, Zhejiang province, China. Distilled and deionized water was produced by a Milli-Q water purification system (Millipore Co., Ltd., Bedford, MA, USA). Reference gallic acid (purity > 98.0%), 5-HMF (purity > 94.0%), morroniside (purity > 98.5%), loganin (purity > 98.0%), sweroside (purity >99.5%), and cornuside scutellarin (purity > 99.5%) were purchased from the National Institute for Food and Drug Control (Beijing, China). All other chemicals and reagents were of HPLC or analytical grade.

### 2.2. Plant Materials

Crude CF was collected from Foping (Shaanxi, China), in which the best quality of the herbs can be guaranteed. CF samples were identified with respect to morphology by College of Life Science, Shaanxi Normal University. Wine-processing CF was obtained according to Pharmacopoeia of the People's Republic of China (2015). Briefly, the crude CF slices were mixed with Chinese rice wine (100 : 15, CF/wine, w/w), and the wine was absorbed into the slices completely. Then, the moistened slices were stir-fried in a metallic pan over a low flame at 120–140°C till they were entirely dried.

### 2.3. Ultrahigh-Performance Liquid Chromatography (UPLC) of the Extracts

#### 2.3.1. Extraction of Samples

Methanol (20 mL) was added to 1.0 g of the powders of crude and wine-treated CF, respectively. Each mixture was then processed in an ultrasonic bath for 5 min. After suction filtration, the filtrate was collected and then 80% methanol was used to make up area weight. Finally, the filtrate was passed through a 0.22 *μ*m millipore syringe filter for UPLC analysis. The contents of six main pharmacodynamic components in CF alcohol extracts were measured quantitatively by the external standard method using the same chromatography conditions as described below.

#### 2.3.2. Chromatographic Conditions

UPLC analysis of phenolic compounds was performed using Shimadzu LC-30AC pumps (Shimadzu Co., Kyoto, Japan), and chromatographic separations were performed on a Shim-pack XR-ODS III column (2.0 mm × 75 mm, 1.6 *μ*m). The mobile phase consisted of acetonitrile (solvent A) and 1% phosphatic acid in water (solvent B) at a flow rate of 0.2 mL·min^−1^. Gradient elution was performed as follows: a linear gradient elution was applied from 2% to 5% solvent A starting from 0 to 3 min; from 5% to 17% solvent A from 3 to 5 min; from 17% to 17.5% solvent A from 5 to 10 min; from 17.5% to 30% solvent A from 10 to 11 min; and from 30% to 60% solvent A from 11 to 15 min. Operating conditions were as follows: column temperature, 30°C; injection volume, 10 *μ*L; and UV-diode array detection at 250 nm. Six biologically active compounds (gallic acid, 5-HMF, morroniside, loganin, sweroside, and cornuside) in the samples were identified by comparing their relative retention times and UV spectra with those of standard compounds and were detected using an external standard method.

#### 2.3.3. Method Validation

The method was validated for linearity, precision, repeatability, stability, and recovery according to the US Food and Drug Administration (USFDA) guidelines [22]. A six-point linearity curve was constructed for each analyte. The calibration curves were run on each analysis day, and the coefficient of determination (*r*^2^) was used to judge linearity. The calibration curves were plotted with the peak area ratio of gallic acid, 5-HMF, morroniside, loganin, sweroside, and cornuside on *y*-axis and concentration on the *x*-axis, and the regression equation was calculated for each curve. The obtained data were submitted to the regression analysis, and correlation coefficients were calculated for these six alcohol extract components using Microsoft Excel. The precision was evaluated using six replicate determinations of a sample, and the relative standard deviation (RSD) was used to evaluate the precision of the analysis method. The repeatability was examined in six replicate samples, and the stabilities of gallic acid, 5-HMF, morroniside, loganin, sweroside, and cornuside were assessed by determining the sample kept at room temperature for different times after preparation (0, 2, 4, 8, 12, and 24 h), which exceeded the routine preparation time of samples. Mean peak areas obtained from the analysis of the stored samples were compared to those obtained from the analysis of freshly prepared samples. Recoveries were tested at low, medium, and high concentration levels (*n* = 6). A mixture containing the analytes including standard at concentrations resulting in the low, medium, and high levels, respectively, were spiked to 3.0 mL of blank sample. The spiked samples were extracted and analyzed according to the procedure described above. Furthermore, the concentrations of gallic acid, 5-HMF, morroniside, loganin, sweroside, and cornuside were calculated using the calibration curves. Recovery was calculated by comparing the determined amounts for extracted samples with the known amounts added.

### 2.4. Development of T2DM

#### 2.4.1. Animals

Kunming male mice (weight 20 ± 2 g) were purchased from the College of Medicine, Xi'an Jiaotong University (Xi'an, China). The mice were housed in standard cages at a constant temperature (22 ± 1°C) and humidity (a relative humidity of 60 ± 5%), under a 12 h light/dark cycle, with free access to food and water. All the animal studies were performed in accordance with the Animal Ethics Committee of Shaanxi Normal University for animal experimentation. All efforts were made to minimize the number of animals and their suffering. Animals were randomly divided into five groups, namely, the diabetes model group (DM), crude products group (CP), wine-processed products group (PP), metformin tablets group (Met), and normal control group (NC). Each group included 10 mice.

#### 2.4.2. High-Fat Diet Streptozotocin-Induced Diabetes

According to the classical method [23], after 3 days of adaptation, 12 mice were fed with a normal diet and other mice with a HFD (60% kcal% fat). After 4 weeks, HFD-fed mice were given STZ at the dose of 40 mg per kg body weight (bw) for 4 consecutive days by intraperitoneal injection. The normal diet mice were given citrate buffer (PH 4.5) equivalent to the STZ. Seventy-two hours after the last injection, mice with fasting blood glucose (FBG) levels greater than 7.8 mmol·L^−1^ were considered as T2DM mice and selected for further experiments.

### 2.5. Improving Effect of Wine-Processed CF and Crude CF for Treating Diabetes

#### 2.5.1. Preparation of Drugs

The powder of CF was set to a daily administration of 30 g for an adult weighing 70 kg [24]. Thus, the calculation of the dose for mice was 3.87 g/kg. We used sonication to get a suspension which contained 3.87 g powder of crude or wine-processed sample and 10 ml 0.4% CMC-Na. Metformin (0.25 g three times a day) was converted to mice's dosage of 70 mg/kg.

#### 2.5.2. Experimental Design

**Figure 1:**
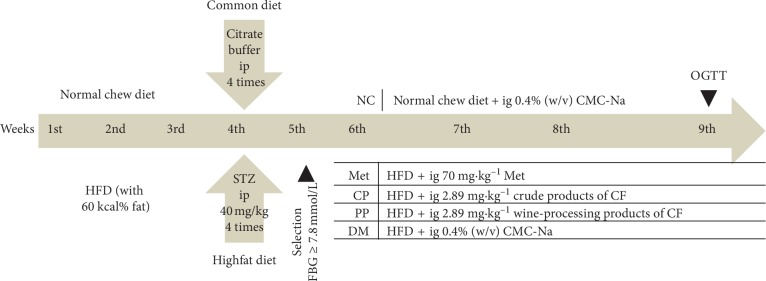
Experimental design. ip, intraperitoneal injection; ig, intragastric administration.

Grouping and treatment protocol is listed in Figure 1. The normal and model groups were given equivalent volumes of 0.4% (w/v) CMC-Na. Other groups were treated with a gastric perfusion of drugs for 28 days (corresponding dosage). Body weight and fasting blood glucose (FBG) of mice were measured once a week during the experimental period. On the last day of the experiment, the mice were made to fast overnight, blood samples were collected via the retro-orbital sinus of mice and separated from serum through centrifugation and were immediately stored at −80°C for biochemical assays. Following the sacrifice of mice via cervical dislocation, liver tissues were carefully excised and washed with ice-cold saline, and portions of each were fixed in 10% formalin (pH 7.4) for biochemical estimations and histological examinations. All experiments using mice were performed in accordance with the National Institute of Health's Guide for the Care and Use of Laboratory Animals and were approved by the university's Institutional Animal Care and Use Committee of Shaanxi Normal University (Xi'an, China).

#### 2.5.3. Oral Glucose Tolerance Test (OGTT)

To study the chronic effect of CF on postprandial glycemic control, the oral glucose tolerance test (OGTT) was performed at week 4. Before the test, mice were fasted for 12 h and the level of blood glucose was measured. The glucose solution was prepared at a concentration of 2 g·kg^−1^ and administered to the mice. Then, blood glucose levels were determined at 30, 60, 120, and 180 min after glucose delivery.

#### 2.5.4. Biochemical Assay

The fasting blood glucose (FBG) level was determined by using one-touch glucometer. The fasting serum insulin (FINS) was determined by the ELISA kit. The insulin resistance index (HOMA-IR) was calculated as FBG (mmol/L) × FINS (*μ*U/mL)/22.5, IR was defined as HOMA-IR > 2.6 [25–27], and the homeostasis model assessment-*β* (HOMA-*β*) was calculated based on the following formula: HOMA-*β* = [(20 *∗* FINS)/(FBG − 3.5)] [28]. All parameters were obtained according to the manufacturer's instructions. Blood samples were stored in a freezer at −80°C until further use.

#### 2.5.5. Assays of Serum

TG, TC, LDL-C, and HDL-C levels were measured using commercially available diagnostic kits. Plasma levels of free fatty acids (FFAs) in all groups were determined by ELISA kits. ALT and AST levels (as biochemical makers for acute liver injury) were assessed using commercially available diagnostic kits. All measurements were carried out according to the manufacturer's instructions of the corresponding kits.

#### 2.5.6. Histological Examination

The livers were fixed in 10% buffered formalin for 24 h and dehydrated with a graded alcohol series (70%, 95%, 100%). Then, the tissues were embedded in paraffin and cut into 4 *μ*m thick sections. Two slices from a same liver were stained with Oil Red O and hematoxylin and eosin (H&E). The slices were observed by using a light microscope.

### 2.6. Statistical Analysis

Statistical analysis was conducted using SPSS software (IBM, USA). Analysis of variance (ANOVA) was in a completely randomized design and SPSS software 17.0 was used. The values were expressed as mean ± standard deviation. All determinations were done at least in triplicate, and all were averaged. A value of *P* < 0.01 was considered statistically significant.

## 3. Results

### 3.1. Validation of UPLC Methodology and Elution Profile

Validation parameters for UPLC are shown in Table 1. The relationship between the peak area ratio and the concentration is linear within the studied concentration range. The correlation coefficients (*r*^2^) obtained from the least square regression analysis of calibration curve was more than 0.9996 for all the analysts. The precision, repeatability, and stability of the method, expressed by the RSD, were lower than 2.2% at each tested sample. These results indicated that the present method has an acceptable degree of precision, repeatability, and stability. Absolute recoveries, obtained for six standards at three different concentration levels, show that extraction recoveries of gallic acid, 5-HMF, morroniside, loganin, sweroside, and cornuside ranged between 98.7% and 100.2%. There were no significant differences at different concentration levels for most of the analytes. These results for method validation suggested that the proposed UPLC method that was developed in our study was reliable for evaluating the alcohol extracts of CF.

**Figure 2:**
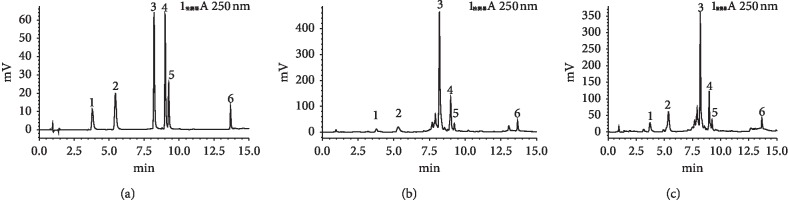
The typical UPLC elution profile of CF extracts, including the control sample (a), the crude extracts (b), and the wine-processed products (c). The first elution peak is gallic acid (1), and the following ones are 5-HMF (2), morroniside (3), loganin (4), sweroside (5), and cornuside (6).

The typical UPLC elution profile of CF extracts, including standard sample, crude extracts, and wine processing, was presented in Figure 2. Clearly, the first elution peak is gallic acid, and the following are 5-HMF, morroniside, loganin, sweroside, and cornuside.

### 3.2. Composition Changes of CF after Wine-Processing

**Figure 3:**
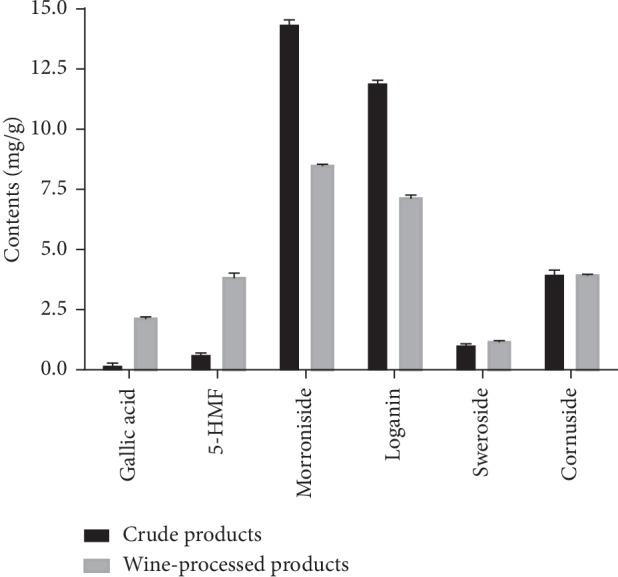
The contents of six compounds in crude and wine-processed CF alcohol extracts.

As shown in Figure 3, the amounts of gallic acid and 5-HMF in CF after wine processing increased more than ninefold and fivefold, respectively, while the amounts of sweroside and cornuside remained stable. In contrast, the yields of morroniside and loganin in wine-treated CF were significantly reduced, 42.9% and 40.3% lower than those in crude CF, respectively. As we expected, wine treatment obviously influences the chemical composition of CF.

### 3.3. Effects of Pre- and Postprocessing of CF on Body Weight and Blood Glucose Levels in STZ/HFD-Induced Diabetic Mice

**Figure 4:**
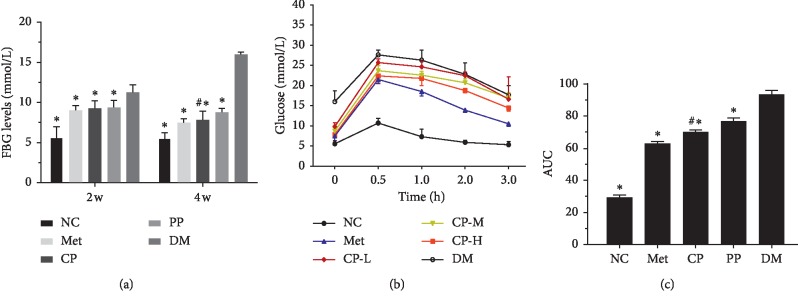
Effect of pre- and postprocessed CF on FBG (a), OGTT (b), and AUC of panel B (c). NC: normal control group; Met: diabetic mice treated with 70 mg kg^−1^ metformin; CP: diabetic mice treated with 2.89 mg kg^−1^ crude CF; PP: diabetic mice treated with 2.89 mg kg^−1^ wine-processed CF; DM: diabetic mice treated with equivalent volumes of 0.4% (w/v) CMC-Na. Values are expressed as mean ± SD of 10 mice in each group. ^*∗*^*p* < 0.01 compared with the DM group and ^#^*p* < 0.01 compared with PP group were considered as very significant difference.

As shown in Table 2, body weight of mice in DM group was significantly lower than that in the NC group during the experiment period (*p* < 0.01). Body weight of diabetic mice that received 4 weeks of CF (either CP or PP) and metformin was increased significantly compared with that of the DM group (*p* < 0.01). Meanwhile, we administered crude and wine-processing CF orally to DM mice to measure the glucose-lowering effects. As shown in Figure 4(a), the FBG level of mice in the DM group was significantly higher than that in the NC group. CF exhibited a positive effect on hypoglycemic activity in diabetic mice, and mice in the CP, PP, and Met groups all showed significantly decreases of hypoglycemic activity compared with that of DM group (*p* < 0.01). It is worth mentioning that compared with PP, CP has more obvious effect (*p* < 0.01). These results indicated that the effect of crude CF was similar with that of Met, which increased the body weight and decreased the FBG level in HFD + STZ-induced diabetic mice.

### 3.4. Oral Glucose Tolerance Test

OGTT was conducted after 4 weeks of CF treatment, in which all the experimental mice were fasted overnight. Blood glucose level increased when the mice were orally given glucose solution (2 g·kg^−1^) and reached the peak within 15∼30 min continuously. While the blood glucose level of the NC mice decreased to its initial level after 3 h, the parameter that remained at a high level in diabetic mice during the OGTT experiments illustrated that the glucose tolerance of DM declines. Treatment with both CP and PP in diabetic mice significantly inhibited the rise in blood glucose levels after oral glucose administration (Figure 4(b)). The overall area under curve (AUC) of glucose was calculated over 3 h, and it is shown that AUC of CP, PP, and Met groups is significantly lower than that of the DM group (Figure 4(c), *p* < 0.01).

### 3.5. Effects of Pre- and Postprocessing of CF on Insulin Sensitivity in STZ/HFD-Induced Diabetic Mice

We further investigated and compared the insulin sensitizing activity of pre- and postprocessing of CF on diabetic mice. As shown in Table 3, FINS level, which could trigger IR, of the DM group was abnormally elevated compared with that of the NC group (*p* < 0.01). However, both crude and wine processing suppressed this trend and lowered the insulin level to the different extents (CP decreased by 24.64%, *p* < 0.01, and PP decreased by 12.32% with no significant difference). HOMA-IR was tested to evaluate the IR in the diabetic mice, which is epidemiologically practical widely used and correlates acceptably (*R* = 0.73–0.88) with the hyperinsulinemic-euglycemic clamp test [29]. Although our primary study outcome was IR, we also examined continuous HOMA-IR and beta-cell function (HOMA-*β*) in secondary analyses. Experimental results showed that, in the presence of CP and PP, the diabetic mice showed reduced HOMA-IR and elevated HOMA-*β*, and these effects were significant in the CP group.

### 3.6. Serum Biochemical Marker Levels

**Figure 5:**
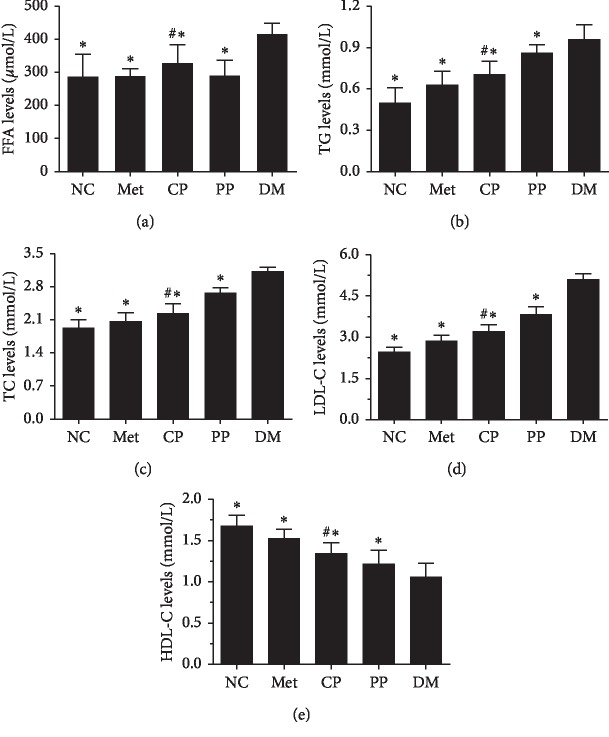
Effects of pre- and postprocessed CF on serum FFA (a), TG (b), TC (c), LDL-C (d), and HDL-C (e) levels in DM mice. NC: normal control group; Met: diabetic mice treated with 70 mg·kg^−1^ metformin; CP: diabetic mice treated with 2.89 mg·kg^−1^ crude CF; PP: diabetic mice treated with 2.89 mg·kg^−1^ wine-processed CF; DM, diabetic mice treated with equivalent volumes of 0.4% (w/v) CMC-Na. Values are expressed as mean ± SD of 10 mice in each group. ^*∗*^*p* < 0.01 compared with the DM group and ^#^*p* < 0.01 compared with PP group were considered as very significant difference.

Diabetes mellitus is one of the most common metabolic diseases, and abnormal lipid metabolism is usually an important determinant of the course and condition of the disease [30]. Previous studies have proved that individuals with higher fasting levels of plasma FFAs were at modestly higher risk of T2DM [29]. To further investigate the ability of pre- and postprocessing of CF on lipid metabolism regulation, lipid metabolic parameters were determined in all experimental groups. As shown in Figure 5, the increased serum levels of TC, TG, LDL-C, and FFAs in DM were significantly (*p* < 0.01) compared with those of NC. However, HDL-C was notably (*p* < 0.01) decreased when the diabetic mice were treated with CP versus DM. The effects of PP on the lipid profile level showed a similar pattern. Moreover, FFAs and TG in the CP group were significantly lower than those in the PP group (*p* < 0.01), which suggested that the CF in crude had greater effects on improving the lipid metabolism disorder and alleviating the liver fatty degeneration.

**Figure 6:**
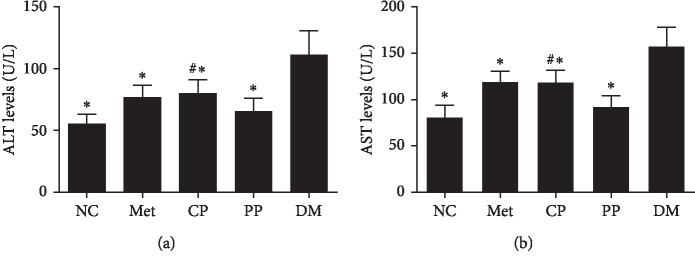
Effects of pre- and postprocessed CF on serum ALT (a) and AST (b) levels in DM mice. NC: normal control group; Met: diabetic mice treated with 70 mg·kg^−1^ metformin; CP: diabetic mice treated with 2.89 mg·kg^−1^ crude CF; PP: diabetic mice treated with 2.89 mg·kg^−1^ wine-processed CF; DM: diabetic mice treated with equivalent volumes of 0.4% (w/v) CMC-Na. Values are expressed as mean ± SD of 10 mice in each group. ^*∗*^*p* < 0.01 compared with the DM group and ^#^*p* < 0.01 compared with PP group were considered as very significant difference.

Some prospective epidemiological studies have demonstrated that AST and ALT, independent of age, alcohol intake, and obesity, were closely associated with the occurrence of T2DM [31, 32]. STZ caused hepatotoxicity in mice, as indicated by increases in serum AST and ALT levels after the DM model established. Rise of serum AST and ALT levels was prevented in Met and both CP and PP-treated mice (Figure 6). However, Met increased the level of AST and ALT compared with PP groups, demonstrating that it causes little damage to the liver. Similarly, CP increased the levels of AST and ALT. While CP had certain toxicity to the liver, PP was found to be safe. Thus, we speculated that the CF processed by wine have effects on reducing the toxicity of TCM.

### 3.7. Histopathological Examination of the Mice Liver

**Figure 7:**
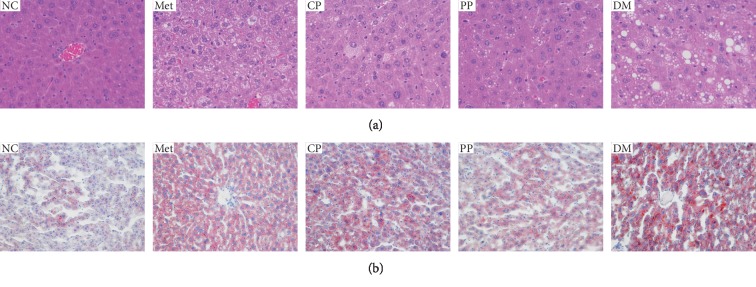
Effects of pre- and postprocessed CF on histopathological changes of liver hepatocytes stained with H&E (a) and Oil red O (b) in DM mice. Images were obtained from each test group. NC: normal control group; Met: diabetic mice treated with 70 mg·kg^−1^ metformin; CP: diabetic mice treated with 2.89 mg·kg^−1^ crude CF; PP: diabetic mice treated with 2.89 mg·kg^−1^ wine-processed CF; DM: diabetic mice treated with equivalent volumes of 0.4% (w/v) CMC-Na.

The liver is the indispensable and key part of insulin action and catabolism and regulates glucose metabolism. T2DM usually induces the injury and damage the function of the liver [33]. Histopathological studies of the liver further provided supportive evidences for the biochemical analysis. According to the result of HE staining (Figure 7(a)) and Oil red O staining (Figure 7(b)), the liver tissue of the NC group was intact, the hepatocytes were normal and regular, and the cytoplasm was homogeneous, and no obvious red lipid droplets were observed. In contrast, DM mice showed severe damage including irregular structure of hepatic lobules, enlarged cell size, many vacuoles in the cytoplasm, and plenty of red lipid droplets in the hepatocytes. The damage of liver tissues was repaired in Met, CP, and PP groups, which showed the basic normal structure of hepatocytes with decreased signs of fatty degeneration, necrosis, and central vein. At the same time, in the liver cells of red lipid droplets, there are different degrees of reduction. It is interesting that PP in the diabetic mice reduced liver lipid deposition and has more remarkable effect than CP. Histopathological analysis gave a perceptual intuition result that PP could be more beneficial to the management of diabetic organ damage and complications compared with CP and Met. These histopathological examination results were in accordance with the plasma parameters.

## 4. Discussion

In this study, an UPLC method was developed to determine six active components in CF, simultaneously. This newly established method is validated as simple, precise, and accurate. It can be used as a valid analytical method for intrinsic quality controls of CF. The contents of each compound between CP and PP varied significantly. Furthermore, the increase of 5-HMF and gallic acid is predominantly due to the decomposition of hexose after heating and the hydrolysis of tannins under high temperature, respectively. Accordingly, PP disintegrated during the heating resulted in a significant reduction of morroniside and loganin, which are the main components of iridoid glycoside with strong cardiac antishock effect. Their reduction may lower the effect of CF on antishock and solid collapse, which is consistent with the view that CP is conducive to the absorption of sweat and solid collapse traditionally. Interestingly, the reasons that the contents of sweroside and cornuside increased slightly after wine processing have yet to be further researched.

The HFD- and STZ-induced diabetic mice were characterized by hyperglycemia, compensatory hyperinsulinemia, elevated triglycerides, and hypertriglyceridemia, which were similar to the features of the later stage of T2DM [34]. CF extracts showed a potential to control postprandial hyperglycemia by *α*-glucosidase inhibition [35]. After adopting Wily wine-processing methods, the treated herbs enhanced blood circulation and accelerated drug delivery [36]. However, for the first time, we showed the comparative study on the antidiabetic activities of crude and wine-processing CF in STZ/HFD-induced diabetic mice. It was found that both of CP and PP could mitigate body weight loss, and some organ weights were restored to the normal level by alleviating hepatomegaly and renal swelling. The body weight loss and the increase of organ index were considered to be the typical T2DM characteristics induced by STZ and HFD [37]. The results indicated that both CP and PP could ameliorate the decrease in body weight and improve the quality of life in diabetic mice. Hyperglycemia is the most important symptom of T2DM [38]. CP could more efficiently reduce FBG and lower the AUC glucose value, which means that CF in crude possesses better curative effect on improving the diabetic mice glucose tolerance.

T2DM is characterized by increased IR, deteriorating *β*-cell function and resulting in chronic hyperglycemia. From the significant increase of the fasting serum insulin (FINS) in the CP and PP groups, especially in CP group, it could be concluded that the crude drug stimulated insulin secretion more effectively in the islets of diabetic mice. In addition, HOMA-IR and HOMA-*β*, most commonly used indexes of IR, further confirmed that the effect of crude CF improving IR is better than that of PP. FFAs, of great significance, can lead to an increase in visceral fat [39]. It has been shown that the continued high levels of blood lipids and FFAs can directly lead to the IR in the liver and peripheral cells [23, 40]. The notable decrease in TG, TC, LDL-C, and FFAs values and the significant increase in HDL-C levels in the CP and PP groups suggested that crude products exhibit better improvement of the abnormal lipid metabolism in diabetic mice. Moreover, the effect of CP is more obvious than that of PP. AST and ALT levels were determined to indicate toxicity of the liver, which plays a vital role in regulating glucose and lipid metabolism through severing insulin-mediated events [41]. Our data and histopathological results suggest that wine products could reduce the toxicity and have protective effects on the liver.

Wine processing is one of the most significant processing methods, which has a profound influence on the prescription and clinical therapeutic effect of TCM. According to our present findings, both crude and wine-processing CF exhibited antidiabetic effect, and in vivo intake for 4 weeks significantly attenuated the hyperglycemia and hyperlipidemia syndromes and improved IR in diabetic mice. Our results suggest that wine processing was recommended when CF was used for protecting the liver; however, crude products should be used as antidiabetic drugs.

*Cornus Fructus* are, undoubtedly, a rare Chinese medicine for hypoglycemia, which has the characteristics of low toxic and several targets. The processing of CF can contribute to the utilization of health and well-being natural products, since it can be an effective new drug for the prevention and treatment of diabetes and liver damage. Our findings positively contribute to a better understanding of the distinctions between CP and PP, which may facilitate the clinical applications of sautéing crude herbal medicine. Such natural resources represent potential agents as new additives for food and pharmaceutical products. [42]

## Figures and Tables

**Table 1:** Validation parameters for UPLC.

Chemical	Calibration curve^†^	Correlation coefficient (r2) (*n* = 6)^‡^	Linear range (*μ*g mL^−1^)	Precision RSD (%)	Repeatability RSD (%)	Stability RSD (%)	Recovery (%)
Gallic acid	*Y* = 3.7 × 10^6^*X* − 1441.3	0.9997	0.00∼0.123	0.7	0.9	2.1	99.3
5-HMF	*Y* = 5.2 × 10^6^*X* + 1336.7	0.9997	0.01∼0.210	0.3	0.9	0.9	98.7
Morroniside	*Y* = 4.9 × 10^6^*X* − 8725.4	0.9999	0.05∼1.050	1.9	2.1	2.1	98.9
Loganin	*Y* = 2.8 × 10^6^*X* + 30041	0.9999	0.05∼1.050	0.5	1.6	0.4	99.2
Sweroside	*Y* = 8.1 × 10^6^*X* + 7581.1	0.9997	0.00∼0.105	2.2	1.7	1.7	100.2
Cornuside	*Y* = 2.5 × 10^6^*X* + 10751	0.9996	0.03∼0.330	1.9	2.2	1.8	98.7

^†^Calibration curves were fitted to the linear regression equation *y* = *ax* + *b*, where “*y*” represents the ratio of the peak areas, “*a*” and “*b*” are constants, and “*x*” is the concentration of the analyzed compounds. ^‡^Number of points in calibration curves.

**Table 2:** Effect on body weight of diabetic mice in pre- and postprocessed CF.

Group	Body weight (g)				
	0 w	1 w	2 w	3 w	4 w
NC	32.89 ± 1.39	33.39 ± 1.23	34.38 ± 1.22^*∗*^	33.82 ± 1.06^*∗*^	33.44 ± 2.48^*∗*^
Met	32.51 ± 2.02	32.82 ± 1.96	32.97 ± 1.92^*∗*^	33.23 ± 1.16^*∗*^	33.24 ± 1.16^*∗*^
CP	32.93 ± 2.26	33.05 ± 1.44	33.32 ± 2.5^*∗*^	33.94 ± 2.34^*∗*^	33.81 ± 1.92^*∗*^
PP	32.35 ± 2.27	33.07 ± 2.35	33.19 ± 2.47^*∗*^	33.37 ± 3.03^*∗*^	33.83 ± 2.01^*∗*^
DM	33.08 ± 1.87	32.77 ± 2.70	30.31 ± 1.67	29.58 ± 1.15	29.72 ± 1.10

The results were shown as mean ± SD of 10 mice in each group. We evaluated ^*∗*^*p* < 0.01 at different weeks which were very significant compared with the DM group.

**Table 3:** Effect on insulin sensitivity of diabetic mice in pre- and postprocessed of CF.

Group	NC	Met	CP	PP	DM
FINS (mIU/L)	11.66 ± 0.76^*∗*^	10.92 ± 1.53^*∗*^	10.42 ± 1.02^*∗*#^	9.39 ± 0.75	8.36 ± 0.72
HOMA-IR	2.86 ± 0.34^*∗*^	3.62 ± 0.62^*∗*^	3.65 ± 1.05^*∗*^	3.66 ± 0.34^*∗*^	5.94 ± 0.75
HOMA-*β*	109.06 ± 8.78^*∗*^	56.14 ± 8.72^*∗*^	51.84 ± 5.02^*∗*#^	35.88 ± 4.09	13.52 ± 1.71

The results were shown as mean ± SD of 12 mice in each group. We evaluated ^*∗*^*p* < 0.01 compared with the DM group and ^#^*p* < 0.01 compared with the PP group and these were considered as significant difference. HOMA-IR = (FBG × FINS)/22.5, HOMA-*β* = (20 × FINS)/(FBG − 3.5).

## Data Availability

The data used to support the findings of this study are available from the corresponding author upon request.
